# Optimizing Dental Care for Adults With Intellectual and Developmental Disabilities: Challenges, Strategies, and Preventative Approaches

**DOI:** 10.7759/cureus.72871

**Published:** 2024-11-02

**Authors:** Caroline Sachse, Rafik Jacob

**Affiliations:** 1 Internal Medicine, University of Florida College of Medicine – Jacksonville, Jacksonville, USA

**Keywords:** barriers to care, dental care, intellectual developmental disorder (idd), lack of health insurance, weight loss and malnutrition

## Abstract

Individuals with intellectual and developmental disabilities (IDD) often face numerous barriers to dental care, leading to higher rates of untreated caries and periodontal disease. This editorial explores the challenges associated with dental care for individuals with intellectual disabilities and suggests methods to enhance their dental care.

Preventive dental care is crucial for maintaining health. Recommendations, mirroring those for the general population, include brushing and flossing teeth twice daily, biannual dental cleanings, and adopting a diet low in acids and added sugars. Specialized toothbrushes with larger handles have been designed to improve daily cleanings and water flossers may be preferred over traditional flossing. Regular observation of independent tooth brushing habits and daily reminders by caretakers contribute to effective plaque removal. Sealants should be placed routinely for additional cavity protection. Patients with IDD may face challenges in practicing ideal preventive care due to oral sensitivity or motor limitations. Caretakers should discuss these challenges to explore feasible adjustments in dental hygiene routines or utensils. When adjustments are not possible, strict dietary control may improve dental outcomes. Sensitivities related to foods may align with dental care sensitivities, necessitating collaboration with occupational therapists and nutritionists to balance nutritional needs while limiting sugary foods.

Dental visits often cause anxiety for individuals with IDD. Studies suggest that conscious sedation, commonly using nitrous oxide, is sufficient for cleanings and minor procedures. Seeking a dentist familiar with caring for patients with IDD can provide additional guidance and care. For those who become edentulous, malnutrition is a common outcome. Dentures are a viable solution, but individuals with IDD may require extra counseling to understand the benefits and risks. Dentures, while effective, pose a choking hazard, which can be mitigated by anchoring with implants or other fixation methods. For those who prefer no reconstructive intervention, dietary adjustments are necessary to meet daily energy and nutritional needs.

Many US insurance companies/systems including Medicaid and Medicare do not sufficiently cover dental cleaning or care, adding an additional barrier to care for patients with IDD. Some dental-specific plans support dental care for patients with IDD, but remain an additional financial burden patients face in protecting their dental hygiene.

## Editorial

Introduction

Adults with intellectual and developmental disabilities (IDD) face numerous barriers, including oral sensitivity, mobility limitations, and inadequate access to dental care. These challenges predispose them to higher rates of untreated dental conditions, such as caries and periodontal disease [1]. The increased prevalence of untreated dental disease significantly impacts overall health. Periodontal disease, resulting from poor oral hygiene, can lead to dental caries and deterioration of the oral bone, ultimately causing tooth loss [2]. Edentulism (loss of teeth) in these individuals is often associated with malnutrition, which may lead to muscle wasting. This editorial seeks to examine the primary obstacles to dental care for individuals with IDD and outline strategies to improve access and quality of care.

Preventive care

Preventive dental care for adults with IDD varies depending on their level of independence, the care provided by caregivers, anatomical differences, and sensory or motor limitations. General dental recommendations for the population also apply to individuals with IDD. Teeth should be brushed twice daily or more frequently when necessary, particularly after consuming sugary or carbohydrate-rich foods. Fluoride-containing dental products and regular flossing help maintain the integrity of interdental spaces and gingival health. Dental cleanings should be conducted at least every six months, and sealants can be applied to prevent cavities. Additionally, making appropriate dietary choices to limit sugar and acid exposure is essential in preventing daily plaque buildup [3-4].

For home oral care, establishing a consistent tooth-brushing routine is key for adults with IDD. Sensitivity to oral care and mobility limitations may severely restrict some patients’ ability to maintain oral hygiene routines. However, modern innovations are improving the oral care toolkit for these individuals. Adaptive toothbrushes and electric toothbrushes can enhance the quality of daily cleaning, while traditional floss can be replaced by water flossers, which may cause less discomfort. Providers should thoroughly explain these recommendations, observe the oral care routine if possible, and offer input for improving preventive care. For those with IDD who are capable of brushing independently, providers should spend additional time reinforcing proper techniques and emphasizing the importance of maintaining this routine [5].

Sedation and care in dental practice

Receiving professional dental care can be a stressful experience for many individuals with IDD. While some dental practices specialize in treating these patients and are well-equipped to manage discomfort and behavioral challenges, access to such practices may be limited. Regardless of the practice’s specialization, conscious sedation can be highly beneficial for patients with IDD who experience anxiety or discomfort during dental care. For more extensive procedures or in complex cases, general anesthesia may be necessary, though the risks of deep sedation should be carefully considered. Conscious sedation using nitrous oxide gas is often effective, and for patients requiring deeper sedation or those who have difficulty tolerating nitrous oxide, oral benzodiazepines can help alleviate procedural anxiety, enabling comprehensive dental care [6].

Diet and nutrition

Dietary restrictions and mindful eating can serve as effective preventive measures against cavities and promote periodontal health. Limiting the intake of sugary and acidic foods, such as soft drinks, citrus fruits, vinegar, and foods with added sugars, is recommended. Consuming less than 15 kg of free sugars per year, or keeping free sugars to less than 10% of daily caloric intake, offers significant protection against dental caries. A protective diet should include a variety of fresh fruits, vegetables, and whole grains while limiting added sugars and fats. These dietary choices have been shown to reduce the risk of dental caries, periodontal infections, and oral malignancies [7].

Adequate fluoride intake further enhances the benefits of dietary modifications in cavity prevention, allowing for greater flexibility in sugar consumption. In the USA, fluoride is commonly found in tap water and fluoride-containing products such as toothpaste and varnishes. These should be used to ensure sufficient preventive fluoride exposure [8]. Additionally, foods that stimulate saliva production, such as nuts, sugar-free gum, and hard cheeses, offer an added protective effect on tooth enamel [7].

It's important to note that nutritional deficiencies are common in individuals with IDD, often due to oral sensitivities to certain textures and flavors, which may limit the quantity and nutrient density of their diet. Addressing malnutrition is crucial for optimizing oral health. This can be achieved through an appropriate diet and vitamin and mineral supplementation to meet energy and nutrient requirements. Essential nutrients often lacking in the diets of patients with IDD, due to oral sensitivities or health challenges, include Vitamins A, C, D, E, B-complex (which can be taken as a multivitamin), iron, and calcium [9]. Optimizing dietary intake, though challenging, is key to preserving oral health by both limiting harmful foods and ensuring the availability of nutrients essential for bone and tooth health.

Edentate individuals

When preventive care is insufficient or inconsistently applied during their younger years, adults with intellectual disabilities (ID) may become edentulous. If this condition remains untreated, a significant consequence is difficulty with eating, which can lead to malnutrition. Proper oral rehabilitation has been shown to effectively address malnutrition in this population [10]. The primary treatment for edentulous patients is the placement of prosthetic dentures. Studies indicate that among a cohort of edentulous individuals with intellectual disabilities, 69.3% did not use dentures, with 9.3% of those possessing dentures choosing not to use them [11]. Notably, none of the patients were denied access to dentures. The study concludes that additional counseling and informed consent should be prioritized to ensure this population receives appropriate dental and nutritional care [10-11].

A separate study evaluating the use of dental implants in adults with ID found that dental reconstruction with implants can be a highly beneficial solution for these patients. However, successful outcomes require proper oral hygiene and preventive care post-implantation. Financial constraints and challenges related to post-operative maintenance are common barriers, particularly due to issues such as sensory sensitivities, motor limitations, bruxism, behavioral difficulties, and gaps in daily care routines [12].

Insurance as a barrier to care

Dental care can be prohibitively expensive for many patients, particularly because U.S. insurance policies rarely cover dental services. This presents a significant barrier to maintaining proper dental hygiene for individuals with IDD. Medicaid requires states to provide dental coverage for children, but there is no mandate to offer dental care to adults. As a result, coverage for preventive dental care and procedures is inconsistent and varies by state, with most states offering minimal or no dental benefits for adults with IDD [13]. Traditional Medicare does not include dental health, while Medicare Advantage and standalone dental plans offer limited coverage, often with high out-of-pocket costs for patients [14-15]. Even commercial insurance plans typically have high co-pays, placing a financial strain on patients.

While some dental-specific insurance plans may cater to patients with IDD, the financial burden remains substantial, and many patients are unable to overcome these barriers. This lack of affordable coverage significantly limits patient access to care, making both preventive measures and necessary treatments relatively unattainable. Although home-based preventive care and nutritional support can be helpful, individuals with IDD benefit greatly from regular dental cleanings, professional treatment, and ongoing education for continued oral health.

Conclusions

Adults with IDD often face distinct challenges, such as heightened sensitivity, limited independence, and mobility constraints, which can make maintaining good oral hygiene and accessing adequate dental care more difficult. Healthcare providers caring for these individuals should communicate effectively with patients, caregivers, and families to emphasize the importance of comprehensive oral care within the context of overall health. Preventive dental care - through consistent tooth brushing, regular cleanings, and appropriate dietary choices - is vital for maintaining both oral health and nutritional balance. For patients who become edentulous, proper guidance on the use of dentures or dental implants can help address malnutrition caused by tooth loss. However, it is important to note that most U.S. insurance policies do not provide sufficient coverage for preventive dental care or procedures, making access to proper care out of reach for many patients.
